# *CCDC40* mutation as a cause of infertility in a Chinese family with primary ciliary dyskinesia

**DOI:** 10.1097/MD.0000000000028275

**Published:** 2021-12-23

**Authors:** Li Liu, Kechong Zhou, Yuxuan Song, Xiaoqiang Liu

**Affiliations:** Department of Urology, Tianjin Medical University General Hospital, Tianjin, China.

**Keywords:** *CCDC40*, hypo-osmotic swelling test, intracytoplasmic sperm injection, infertility, primary ciliary dyskinesia

## Abstract

**Trial design:**

Primary ciliary dyskinesia (PCD) is a genetical disease that inherited in an autosomal-recessive way. Its clinical manifestations (such as male infertility) are mainly caused by defects of motion-related cilia that encoded by mutated genes. Although some mutation has been verified, a number of mutations of PCD remain elusive. The main purpose of this study is to identify mutant genes in a Chinese family with PCD, and to verify the safety and effectiveness of intracytoplasmic sperm injection (ICSI) of infertility caused by PCD.

**Methods:**

Imaging examination was used to exclude pulmonary inflammation and visceral translocation. Semen analysis was used to assess the quality of the proband's sperm. Transmission electron microscopy (TEM) was conducted to assess the ultrastructure of flagella and cilia. Targeted next generation sequencing and Sanger sequencing and qPCR (real-time quantitative polymerase chain reaction detecting system) were applied to identified mutation of Chinese Family suspected of having PCD. Viable sperm were selected by hypo-osmotic swelling test (HOST) for ICSI.

**Results:**

We report 2 novel mutations in *CCDC40* gene (c.1259delA and EX17_20 deletion) resulted in immobility of sperm and infertility of the proband. These mutations were confirmed in the proband's sister (heterozygous) and his parents (recessive carrier) by Sanger sequencing and qPCR. All the spermatozoa from the proband were immotile. Ultrastructural defects were found in flagella and cilia of proband and his sister. Viable sperms were selected by HOST for ICSI and fertilized 9 of 21 eggs. Two frozen embryos were transplanted and a healthy 3500 g boy was delivered at 40 + 4 weeks’ gestation. And then, we summarized the genes related to PCD and the mutant sites of *CCDC40* gene.

**Conclusion:**

We reported 2 novel mutants in *CCDC40* gene (c.1259delA and EX17_20 deletion), which could be candidates for genetic diagnosis in PCD patients. The combination of targeted next generation sequencing and Sanger sequencing may be a useful tool to diagnose PCD. ICSI is a considerable method in treatment of infertility caused by PCD.

## Abstract 2

Supplemental Digital Content is available in the text

## 1 Introduction

**Figure 1:**
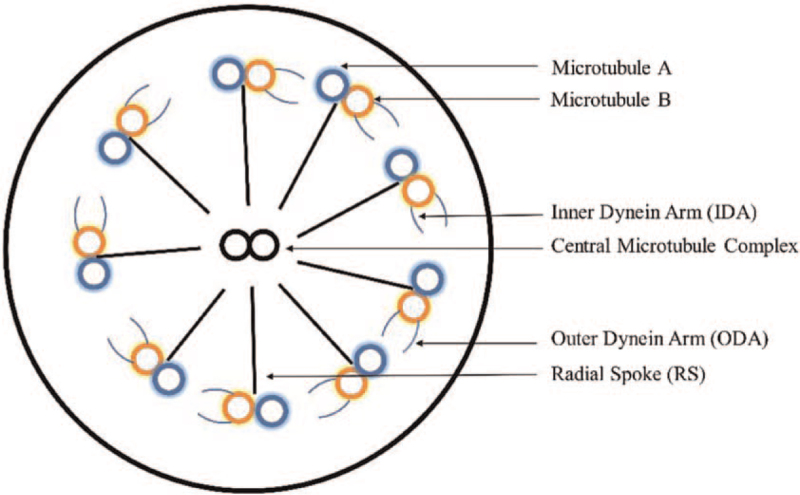
Diagrammatic representation of normal ciliary ultrastructure adapted from Lobo et al.^[6]^

Primary ciliary dyskinesia (PCD) is an autosomal-recessive heterogeneous syndrome (prevalence 1:10,000 to 1:40,000 births) acknowledged by disorder of motile cilia in cells such as epithelial airway cells and spermatozoa.^[1–4]^ This syndrome include chronic sinusitis, neonatal respiratory distress, bronchiectasis, infertility, and situs inversus.^[5]^ In PCD, >30 protein-coding genes are involved in the structure or assembly of the axoneme and cytoskeleton in cilia.^[6,7]^ The sperm flagellum is an evolutionarily conserved organelle. It has motile functions. Axoneme is an intrinsic structure of sperm and flagellum, which can mediate motility (powered by motility dynein arms [DA]).^[8]^ The normal structure of axoneme is “9 + 2” pattern (Fig. 1), which are powered by motility DA. The sperm flagellum defects have been linked to several human diseases.^[9]^ PCD, previously known as immotile cilia syndrome, is the first human genetic disorder associated with cilia dysfunction that affects flagellum abnormalities.^[4]^ The diagnosis of PCD mainly depends on recognizing the characteristic clinical phenotype and interpreting diagnostic tests.^[10]^ Recently, significant progress has been made in genetic analysis and electron microscopy examination. More gene mutations and microstructural abnormalities of PCD have been found.^[11,12]^ However, PCD is a highly heterogeneous disease, and the clinical manifestations vary greatly among patients even in the same family. So PCD still needs to be further clarification.

The treatment of PCD is mainly to control symptoms and long-term surveillance, such as the application of antibiotics to prevent and treat respiratory infections. It has been reported that about 100% of male PCD patients will have infertility.^[10]^ In the past, such infertile patients (complete loss of sperm motility) were unable to conceive naturally. Therefore, artificial assisted reproduction technology (such as intracytoplasmic sperm injection, ICSI) is usually of great help to male patients with PCD.^[2]^ ICSI have allowed some of these individuals to become fathers using their own spermatozoa, using modified hypo-osmotic swelling test (HOST) to select immotile spermatozoa.^[13]^ Here, we report a 23-year-old man diagnosed as PCD in a nonconsanguineous family having 2 novel mutant allele in *CCDC40*.

The study was approved by the Ethics.

In addition, our study was permitted by Committee of Tianjin Medical University General Hospital. All recruited participants signed informed consent before being enrolled in our study.

## 2 Case report

### 2.1 The proband (III-5)

**Figure 2:**
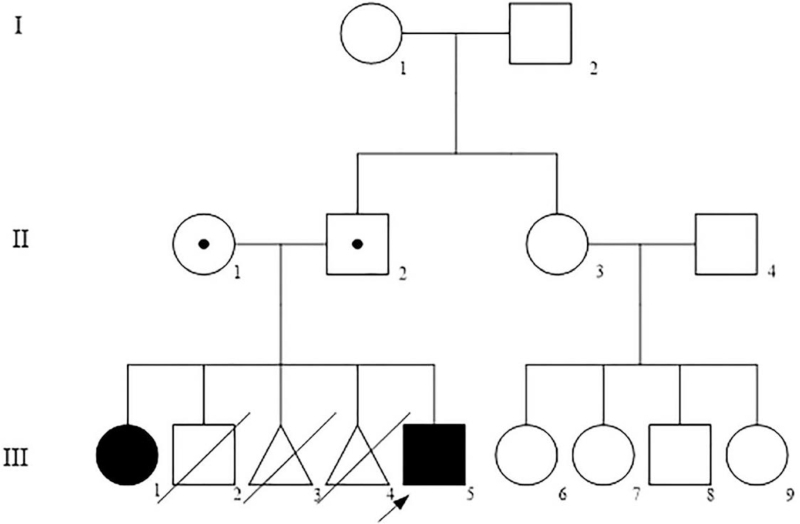
Pedigree structure of the family with primary ciliary dyskinesia.

**Figure 3:**
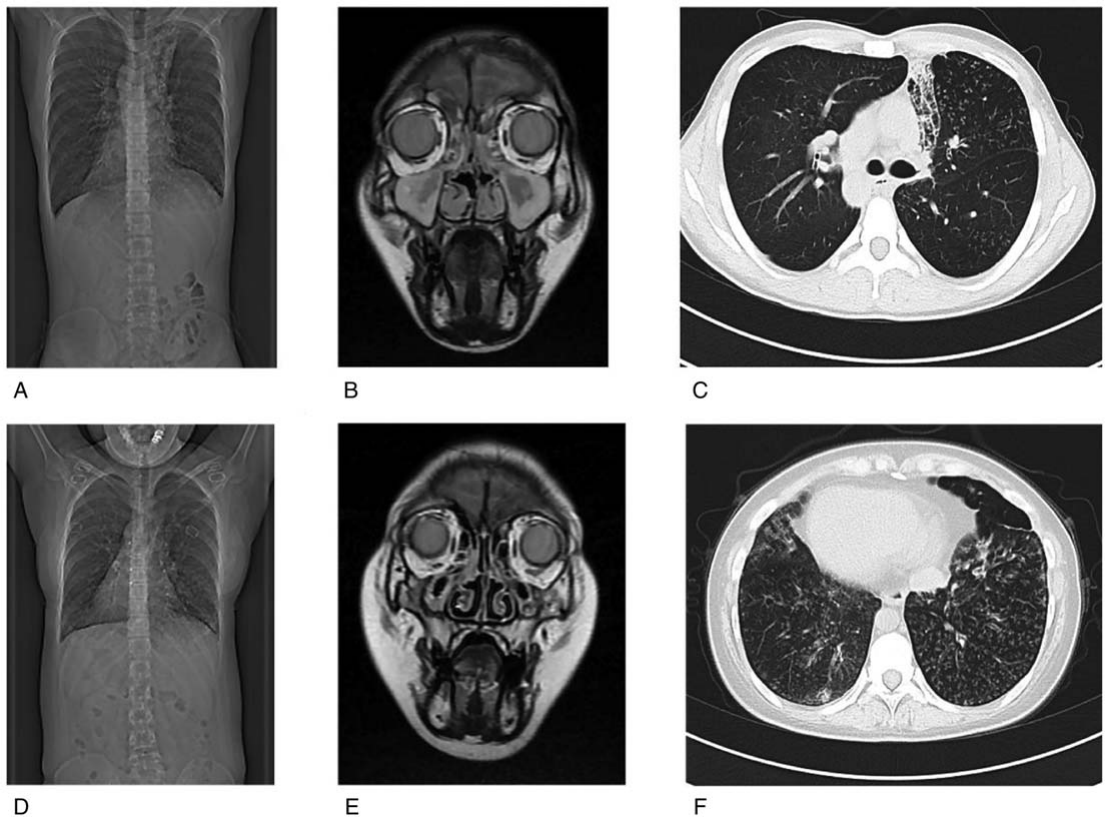
The radiological examination of Proband (IIII-5), (A) CT showed situs inversus. (B) MRI of paranasal sinus shows bilateral ethmoid sinusitis and maxillary sinusitis. (C) HRCT of chest shows bronchitis and bronchiolitis. The radiological examination of Proband's sister (III-1), (D–F) the same but more severe imaging appearance as the proband. CT = computed tomography, HRCT = high-resolution computed tomography, MRI = magnetic resonance imaging.

**Figure 4:**
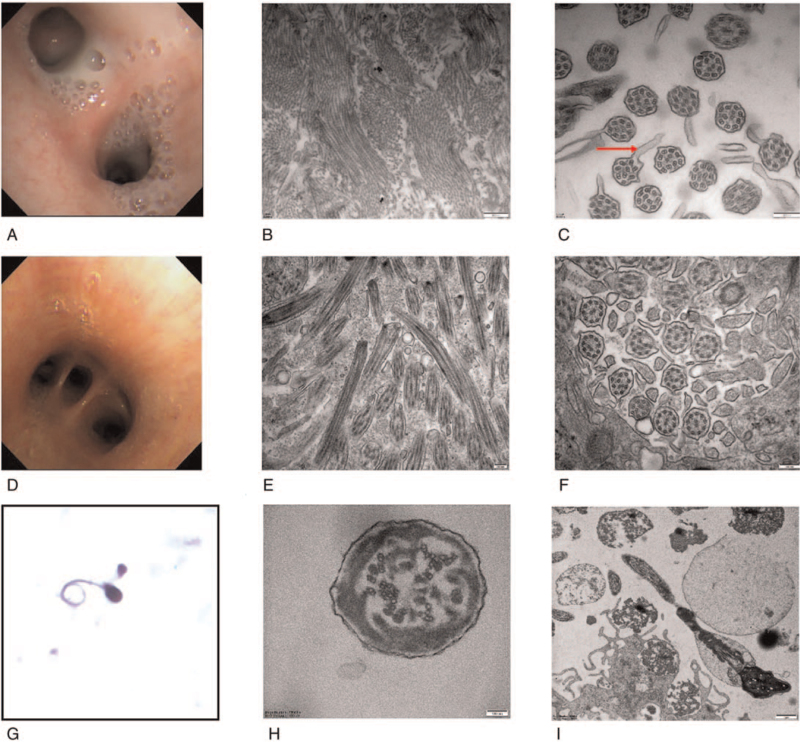
(A) Bronchoscopy examination of the proband's elder sister (III-1): a large number of purulent secretions are secreted by the tracheal carina. (B) Several microtubules in vertical section of the proband's elder sister. (C) The middle section of bronchial mucosa cilia electron microscopy of the proband's elder sister; disorder of DA, microtubular doublets structure and oval synapses (red arrow). (D) Bronchoscopy examination of the proband (III-5): a small amount of purulent secretion is secreted by the tracheal carina. (E) Several microtubules in vertical section of bronchial mucosa cilia electron microscopy of the proband (III-5). (F) The middle section of bronchial mucosa cilia electron microscopy of the proband (III-5); disorder of DA, microtubular doublets structure with some tiny oval synapses. (G) Optical microscope of Diff-quick stained sperm from the PCD patient indicated the curly tail of sperm. (H) Cross-section of the axoneme from the sperm flagellum; abnormal quantities and disorganization of ODFs arrangement; central microtubule pair get lost, decreased in number with perturbed peripheral microtubular doublets structure and radial spokes, dynein arms are beyond recognition. Excess fibrous sheaths are observed. (I) Vertical section of a sperm, the heads are normal while the flagellum is flexural and encircled with the plasma membrane, notice the disarrangements of the mitochondria. DA = dynein arms, PCD = primary ciliary dyskinesia.

The proband (III-5) is a 21-year-old non-smoker man with chronic cough since childhood from a Chinese family (Fig. 2). His parents are nonconsanguineous. He was referred to the Centre for Reproductive Medicine (Tianjin Medical University General Hospital), after 1 year of history of primary infertility (married for 1 year, cohabitation, normal sex life). The sperm analysis manifested a severe oligozoospermia. Multiple semen analyses showed that the sperm density was <1 × 10^6^/mL, and there was no evidence of either non-forward or forward movement (the activity was 0). Normal sperm rate was 0% (most were tail deformities). HOST showed that the sperm survival rate was 52%. The Chromosome karyotype of the proband is 46, XY. No microdeletions were detected at 6 sites (SY84, SY86, SY127, SY134, SY254, SY255). Hormone level test was normal. Paranasal sinus magnetic resonance imaging (MRI) showed bilateral maxillary sinusitis and ethmoid sinusitis (Fig. 3B). Chest computed tomography (CT) showed bronchiectasis and total situs inversus (Fig. 3A). High-resolution computed tomography (HRCT) of chest shows bronchitis and bronchiolitis (Fig. 3C). Bronchoscopy showed extensive hyperemia and edema in the mucosa of the proband. And there is a large number of purulent secretions (Fig. 4D). Abnormal spermatozoa cilia ultrastructure (disordered arrangement, reduction, DA loss) was found by transmission electron microscopic (TEM) analysis (Fig. 4H, I). Broncho-cilia electron microscope (III-5) showed ultrastructural defects (ODA, outer dynein arms; IDA, inner dynein arms and axonal tissue disorders) in the microtubules of 9 + 2 cilia (Fig. 4E, F).

From the proband's medical history and imaging examination, we speculated that the proband may have PCD. Detailed medical history revealed that the sister (III-1) of the proband also had similar clinical manifestations (chronic cough, infertility). Therefore, we conducted bronchial ciliary electron microscopy examination on the probands and his sister, and identified the chromosomal mutation sites of the patients by gene sequencing.

### 2.2 Targeted next generation sequencing and Sanger sequencing

Targeted next-generation sequencing was used to detect mutations of the proband. Roche NimbleGen custom sequence capture human array was used for all flanking sequences and 20 PCD exons (Table S1, Supplemental Digital Content, http://links.lww.com/MD2/A758). Routine Sanger sequencing and quantitative polymerase chain reaction (qPCR) were conducted to confirm supposed mutations in the family members (II-1, II-2, III-1, and III-5).

**Figure 5:**
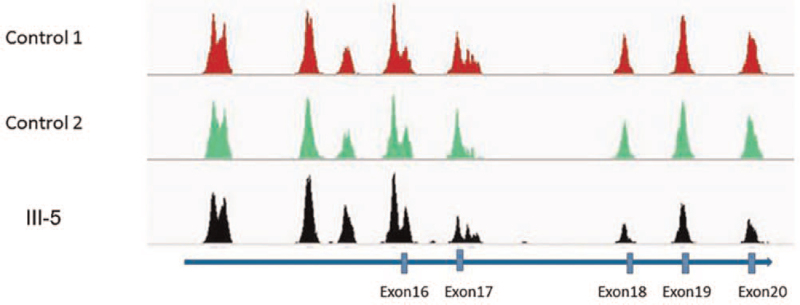
The deletion (EX 17_20del) of the proband.

**Figure 6:**
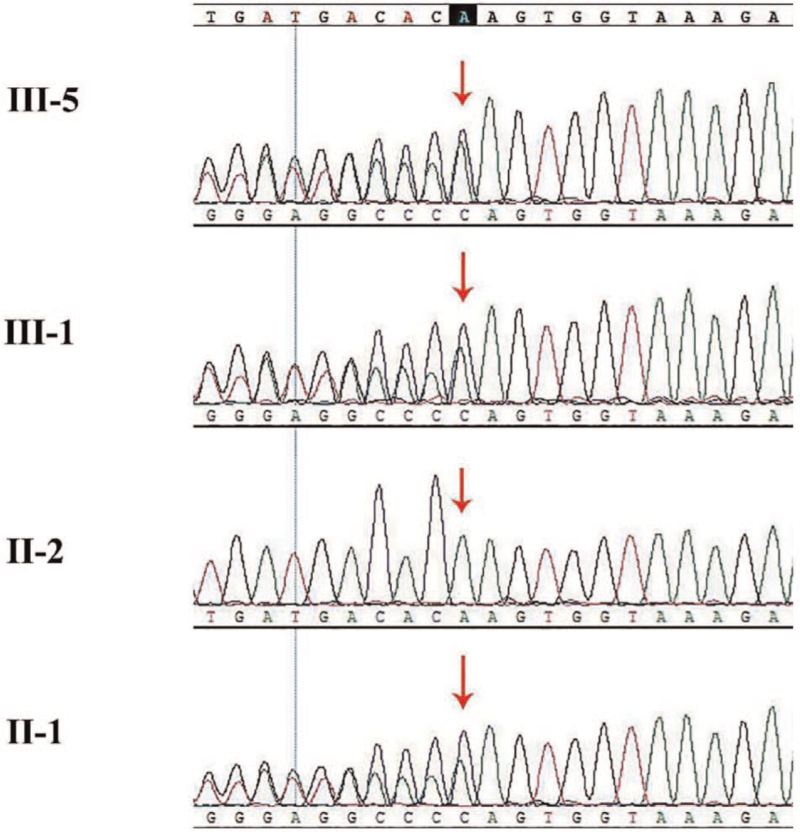
Analysis of *CCDC40* mutation in the family. The deletion (c.1259delA) was validated by Sanger sequencing in the mother (II:1), and inherited to both son (III:5) and daughter (III:1). (The primers: F: 5′-ACCACCTGGCACTACTTCAG-3′, R: 5′-ATACAAGTTGACGCCACCCA-3′).

**Figure 7:**
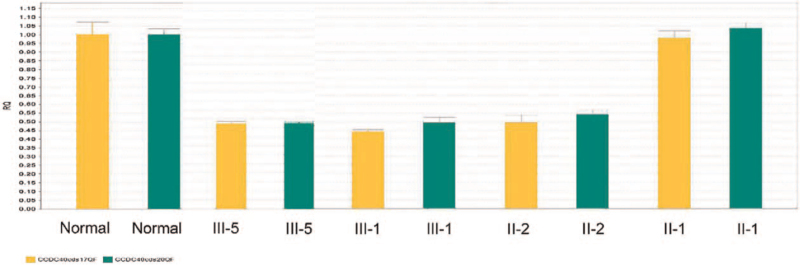
A large deletion (EX17_20del) was confirmed by qPCR in the father (II:2), and inherited to both son (III:5) and daughter (III:1). (The primers: cds17 of CCDC40 F5′-TGCATCTCTTCTACATGCAGAA-3′, R 5′-TCAAACAGGGCAATGTCTTCTT-3′; cds20 of CCDC40 F5′-AACAAGACCACCAAATACTTCAA-3′, R 50- TGTTGGTGATGAGCTCGTTGAC -3’).

Two novel mutations were detected: a mutation in exon 8 (c.1259delA, inherited from his mother), leading to truncated protein, possibly. An EX17_20 deletion (inherited from his father, Fig. 5). We used Sanger sequencing (Fig. 6) and qPCR (Fig. 7) to confirm the mutations in the family members (II-1, II-2, III-1, and III-5).

### 2.3 ICSI treatment of the proband

The proband received 1 cycle of ICSI treatment after genetic counseling. Patients were informed of the genetic risk of ICSI and were given informed consent. The proband’ wife is 29 years old. The long program of reproductive medicine center of Tianjin medical university general hospital was adopted to promote the treatment of ICSI. The sperm of the proband were selected by HOST.

A total of 21 oocytes were obtained, and all viable sperm were screened by HOST for ICSI. There were 9 normal fertilized oocytes, 8 embryos, and 6 high-quality embryos. In June 2017, 2 frozen embryos were transplanted without giving birth, and in August 2017, 2 frozen embryos were transplanted with clinical pregnancy. In September 2017, ultrasound showed an intrauterine pregnancy sac with a yolk sac visible. In May 2018, 40 + 4 weeks of gestation, a baby boy was born naturally, 3500 g, healthy. Until now (July 2019), the baby has not shown any respiratory symptoms, and the chest X-ray indicates that the viscera are in normal position.

### 2.4 The proband’ sister (III-1)

She has been tested though chest CT and sinus MRI same as the proband. The manifestations of her chest CT (Fig. 3D, F) were total situs inversus, bronchiectasia, tree-in-bud sign, bronchial wall thickening, and centrilobular nodules in both lung lobes, which is heavier than the proband. Her paranasal sinus MRI (Fig. 3E) showed lighter pansinusitis than the proband. Bronchoscopy examination showed more purulent secretions than the proband (Fig. 4A). Bronchial ciliary electron microscopy showed that disorder of DA and oval synapses (as shown by the red arrow) (Fig. 4B, C). Chromosome karyotype of her chromosome is 46, XX.

## 3 Discussion

Primary ciliary dyskinesia (PCD, MIM 244400) is autosomal and x-linked recessive disorder. Most of its clinical symptoms are caused by anomalies in the axoneme, that is, the core cytoskeletal structure from flagella and cilia. High complexity of the defects involved in ciliary movements result in extremely heterogeneous clinical manifestations of PCD.^[14]^ More than 200 genes code for ciliary components were verified, and any mutation of these genes can lead to the ultrastructural defects of the cilia.^[15]^ It's the reason why so high genotypic and phenotypic heterogeneity between PCD patients. Therefore, we need to constantly discover new gene mutations diagnosed as PCD to enrich our diagnostic database and thus prepare for individualized treatment. To date, >40 related genes have been identified (Table 1),^[5,6,16]^ most of which are related to ODAs and/or IDAs. In this study, we used targeted gene-based NGS that is rapid and cost-effective method to identify the candidate mutation in patients with PCD. We investigated a Chinese proband with PCD and identified 2 novel mutations in the *CCDC40* gene (a frameshift mutation c.1259delA and an EX17_20 deletion) from a Chinese family; and then, we confirmed these 2 mutations by Sanger sequencing in the family members. Finally, we confirm that the frameshift mutation (c.1259delA) and an EX17_20 deletion, were inherited from his mother and father, respectively. Therefore, we believe that the combination of targeted next generation sequencing and Sanger sequencing can be economically and effectively to diagnose PCD.

**Table 1:** Mutations in the genes that cause human PCD.

PCD locus	Human gene	Human chromosomal location	Ciliary ultra-structure defect	MIM#
CILD1	DNAI1	9p21-p13	ODA defect	604366
CILD2	DNAAF3(C19ORF51)	19q13.42	ODA + IDA defect	614566
CILD3	DNAH5	5p15.2	ODA defect	603335
CILD4	NA	15q13.1-q15.1	NA	608646
CILD5	HYDIN	16q22.2	Normal, very occasionally CA defects	608647
CILD6	TXNDC3(NME8)	7p14-p13	Partial ODA defect (66% cilia defective)	610852
CILD7	DNAH11	7p21	Normal	611884
CILD8	NA	15q24-q25	ODA defect	612274
CILD9	DNAI2	17q25	ODA defect	612444
CILD10	DNAAF2(KTU)	14q21.3	ODA + IDA defect	612518
CILD11	RSPH4A	6q22.1	Mostly normal, CA defects in small proportion of cilia	612649
CILD12	RSPH9	6p21.1	Mostly normal, CA defects in small proportion of cilia	612650
CILD13	DNAAF1(LRRC50)	16q24.1	ODA + IDA defect	613193
CILD14	CCDC39	3q26.33	IDA defect + microtubular disorganization	613807
CILD15	CCDC40	17q25.3	IDA defect + microtubular disorganization	613808
CILD16	DNAL1	14q24.3	ODA defect	614017
CILD17	CCDC103	17q21.31	ODA + IDA defect	614679
CILD18	HEATR2	7p22.3	ODA + IDA defect	614874
CILD19	LRRC6	8q24	ODA + IDA defect	614935
CILD20	CCDC114	19q13.32	ODA defect	615067
CILD21	DRC1	2p23.3	Alterations in the nexin-dynein	615294
CILD22	ZMYND10	3p21.31	ODA + IDA defect	615444
CILD23	ARMC4	10p12.1-p11.23	ODA defect	615451
CILD24	RSPH1	21q22.3	Central microtubule complex and radial spoke defects	615481
CILD25	DYX1C1	15q21.3	ODA + IDA defect	615482
CILD26	C21orf59	21q22.1	ODA + IDA defect	615500
CILD27	CCDC65 (DRC2)	12q13.12	Mostly normal, CA defects in small proportion of cilia	615504
CILD28	SPAG1	8q22	ODA + IDA defect	615505
CILD29	CCNO	5q11.2	Ciliary a/oligoplasia	615872
CILD30	CCDC151	19q13.32	ODA defect	616037
CILD32	RSPH3	6q25.3	Mostly normal, CA defects in small proportion of cilia	616481
CILD33	GAS8	16q24.3	NA	616726
CILD34	DNAJB13	11q13.4	Mostly normal, CA defects in small proportion of cilia	617091
CILD35	TTC25	17q21.2	ODA defect	617092
CILD36	PIH1D3	Xq22.3	ODA + IDA defect	300991
CILD37	DNAH1	3p21.1	NA	617577
CILD38	CFAP300	11q22.1	ODA + IDA defect	618063
CILD39	LRRC56	11p15.5	Normal	618254
CILD40	DNAH9	17p12	ODA defect	618300
CILD41	GAS2L2	17q12	Normal	618449
NA	MCIDAS	5q11.2	NA	614086
NA	DNAH8	6p21.2	NA	603337

MIM#, Online Mendelian inheritance in man (OMM) (http://www.ncbi.nlm.nih.gov/omim) is a continuously updated catalog of human genes, genetic disorders and traits, with particular focus on the molecular relationship between genetic variation and phenotype expression. CA = central apparatus, IDA = inner dynein arms, NA = not available, ODA = outer dynein arms, PCD = primary ciliary dyskinesia.

At least 12% of microtubular disorganization defects are correlated with IDAs, and they are mainly caused by mutations in *CCDC40* (MIM 613808).^[11]^*CCDC40* is an evolutionarily conserved coiled coil domain-containing protein and mutation of *CCDC40* results in cilia will reduce the ranges of motility. Becker-Heck et al^[11,17]^ stated that the human *CCDC40* mapped to chromosome 17q25.3 and the protein contains 1142 amino acids. They found that there was an altered beating pattern in all analyzed samples. Respiratory cilia from affected individuals exhibited markedly reduced beating amplitudes. The cilia appeared rigid with fast movements. They further identified the molecular characterization of this process, and found that CCDC40 protein plays a key role in correct assembling distinct coiled-coil domain-containing proteins, including GAS11-containing DRC and DNALI1-containing IDAs, all of them can eventually regulate the movement of a cilia.^[11,17]^ In addition, *CCDC40* is necessary for proper interconnections among microtubules or serves as a docking domain. Until now, of and its, >30 disease-causing *CCDC40* mutations involvement in PCD have been identified (Table 2).^[11,17]^

**Table 2:** CCDC40 mutations in primary ciliary dyskinesia.

Origin	The number of cases	DNA change	Location	Protein change	Reference
Germany	2	c.248delC	Exon3 + Exon3	p.Ala83Valfs82X	^[17]^
Germany	1	c.1315C>T	Exon8 + Exon 8	p.Gln439X	^[17]^
Pakistan	2	c.1527_1558del	Exon10 + Exon10	p.Asp510Serfs22X	^[17]^
Austria	1	c.1971C>T	Exon12 + Exon12	p.Gln651X	^[17]^
Germany	1	c.3129delC	Exon19 + Exon19	p.Phe1044Serfs35X	^[17]^
Germany	1	c.248delC + c.778del	Exon3 + Exon5	p.Ala83Valfs82X+ p.Glu260Argfs25X	^[17]^
Denmark	1	c.248delC + IVS11-2A>G	Exon3 + Exon12	p.Ala83Valfs82X + splicing	^[17]^
Germany	1	c.248delC + c.1810C>T	Exon3 + Exon12	p.Ala83Valfs82X + p.Gln604X	^[17]^
Denmark	1	c.248delC + c.2824-2825insTGT	Denmark	1	^[17]^
Yugoslavia	1	c.960C>T + c.C2440T	Exon7 + Exon14	p.Arg321X + p.Arg814X	^[17]^
Hungary	3	c.1366C>T + del	Exon9 + del	p.Arg449X + del	^[17]^
Germany	1	c.2824_2825insTGT +	Exon17 + Exon19	p.Arg942MetinsW + p.Gln1041fs36X	^[17]^
		c.3128_3130delC			^[17]^
Germany	1	c.248delC + n.d.	Exon3 + n.d.	p.Ala83Valfs82X + n.d.	^[17]^
N. Europe (UK)	1	c.2712-1G>T	Intron16 + Intron16	Essential splice site	^[11]^
N. Europe (UK)	1	c.2712-1G>T	Intron16 + Exon 17	Essential splice site	^[11]^
Pakistan	1	c.1415delG	Exon 9 + Exon 9	p.Arg472fs3X	^[11]^
Pakistan	1	c.1006C>T	Exon 7 + Exon 7	p.Gln336X	^[11]^
N. Europe (UK); N.	16	c.248delC	Exon 3 + Exon 3	p.Ala83Valfs84X	^[11]^
Germany	1	c.248delC + n.d.	Exon3 + n.d.	p.Ala83Valfs82X + n.d.	^[11]^
S. Europe (Turkish)	1	c.3175C>T	Exon 19 + Exon 19	p.Arg1059X	^[11]^
Africa (Moroccan)	1	c.1464delC	Exon 10 + Exon 10	p.Ile488Ilefs19X	^[11]^
N. Europe (Belgian)	1	c.248delC + c.687delA	Exon 3 + Exon 5	p.Ala83Valfs84X + p.Pro229Profs58X	^[11]^
N. Europe (USA)	1	c.2440C>T	Exon 14 + Exon 14	p.Arg814X	^[11]^
N. Europe	1	c.961C>T	Exon 7 + Exon 7	p.Arg321X	^[11]^
N. Europe (USA)	1	c.940-2A>G + c.344delC	Intron 6 + Exon 3	Essential splice site +p.Pro115Argfs52X	^[11]^
N. Europe (USA)	1	c.248delC + c.961C>T	Exon 3 + Exon 7	p.Ala83Valfs84X + p.Arg321X	^[11]^
N. Europe (USA)	1	c.1345C>T + c.2712-1G>T	Exon 9 + Intron 16	p.Arg449X + essential	^[11]^
China	1	c.2609G>A	nd	p.R870H	^[18]^
China	3	EX17_20del	EX17_20/CDS17_20	n.d.	The present
China	3	c.1259delA	EX8/CDS8	p.Val421TrpfsX2	The present

Del = deletion, fs = frame shift, ins = insertion, IVS = inversion, n.d. = not determined.

Male PCD patients often have comorbid infertility. Because their spermatozoa are immotile, mostly. As is known to all, immotile spermatozoa are unable to fertilize via conventional in vitro fertilization (IVF), fertilization can be achieved with ICSI technique.^[19,20]^ Ebner et al^[21]^ target oocytes with Ca2+-ionophore to restore the sperm motility of theophylline-resistant PCD patients, showed an unusual but effective way to treat infertility. It also indicates that ICSI is an effective way to treat PCD-related infertility. According to the study of Esteves et al,^[22]^ ICSI using spermatozoa extracted from testis (TESE-ICSI) is better than ICSI with ejaculated spermatozoa (EJ-ICSI) for PCD. But there was no difference in the fertilization rate and pregnancy rate between TESE-ICSI and EJ-ICSI. The biochemical pregnancy of ICSI is significantly related with the morphology and vitality of sperm selected to fertilize. Therefore, the key to successful pregnancy after ICSI for patients with PCD is the selection of viable sperms.^[23]^ The HOST is a simple, reliable, and non-damaging technique method recommended by WHO for selecting viable sperm. In the process of treating this patient, we used the HOST method (reduced the low permeability expansion time from 5 minutes to 10 seconds) to screen out the viable ejaculated spermatozoa. Ultimately, the proband succeed in having a healthy baby of their own. This is consistent with previous reports by many researchers demonstrating that ICSI with HOST is an effective tool to select viable spermatozoa and increase the fertilization rate.^[19,24,25]^ Moreover, since PCD is an autosomal recessive genetic disease, children born with ICSI will almost never have PCD as long as the spouse of the patient does not carry the relevant disease-causing gene.

In conclusion, in the last few decades, genetic studies of PCD have uncovered a lot of important ciliary genes. The identification of these genes will lead us to a deeper understanding of the molecular mechanisms involved in the assembly and function of cilia and the pathway. And these findings could allow us to use targeted next generation sequencing and Sanger sequencing to diagnose PCD faster and more efficiently. However, due to the large number of genes involved in this disease, only a few have been confirmed so far. Therefore, we also need to combine full exon sequencing to find new pathogenic genes, so as to enrich our pathogenic gene pool. For patients with PCD combined infertility, ICSI with HOST can also be very effective in helping them have their own healthy children.

## 4 Conclusions

We reported 2 novel mutants in *CCDC40* gene (c.1259delA and EX17_20 deletion), which could be candidates for genetic diagnosis in PCD patients. The combination of targeted next generation sequencing and Sanger sequencing is a useful tool to diagnose PCD. ICSI is a considerable method in treatment of infertility caused by PCD.

## Acknowledgments

The authors sincerely thank Yongjiao Yang (Tianjin Medical University General Hospital) and Shangren Wang (Tianjin Medical University General Hospital) for the help and support of the study.

## Author contributions

**Conceptualization:** Li Liu.

**Data curation:** Li Liu, Kechong Zhou.

**Formal analysis:** Li Liu, Kechong Zhou.

**Funding acquisition:** Xiaoqiang Liu.

**Investigation:** Kechong Zhou, Yuxuan Song.

**Methodology:** Kechong Zhou, Yuxuan Song.

**Resources:** Yuxuan Song.

**Software:** Xiaoqiang Liu.

**Supervision:** Xiaoqiang Liu.

**Writing – original draft:** Li Liu, Kechong Zhou.

**Writing – review & editing:** Li Liu.
